# 

**Published:** 1914-05-15

**Authors:** 


### ANNOUNCEMENTS.

### Tennessee State Dental Association.

The 47th annual meeting of this society will be held in Chattanooga, June 25 to 27, 1914. Especial preparations are being made for a good programme, and it is hoped there will be a large attendance of the members, and such other dentists as may be interested will be welcomed. The time and the place ought to bring out a large attendance and assure a good meeting. Let every body come and contribute something.

C. Osborn, Rhea, Secy.

Nashville.

### Officers Michigan Dental Society.

At the annual meeting held in Detroit, April 9 to 11, the following officers were elected for the ensuing year:

President, Dr. Charles A. Burbridge, of Grand Rapids; Vice President, Dr. George C. Bowles, of Detroit; Secretary, F. Ward Howlett, of Jackson; Treasurer, Edwin J. Chamberlain, of Grand Rapids. Dr. George F. Burke, of Detroit, and Dr. A. C. Thompson, of Port Huron, were elected members of the Executive Council.

A resolution was adopted to ask the State Legislature for the appointment of a public lecturer on Oral Hygiene.

A resolution was also adopted requesting the Council to take such measures as were practicable for assisting local and affiliated district societies in providing better programmes for their meetings.

The society received a very cordial invitation to hold its next meeting in Battle Creek, but this will be determined by the Executive Council.

### Northern Ohio Dental Association.

The Fifty-seventh Annual Session of this Association will be held in Cleveland, Thursday, Friday and Saturday, June 4, 5 and 6, 1914, at the Wigmore Coliseum. All ethical dentists are cordially invited. A good programme and the usual good time is promised.

C. D. Peck, Sec'y,
Sandusky, O.

### Michigan State Board.

The next meeting of the Michigan State Board of Dental Examiners will be held at the Dental College, Ann Arbor, commencing Monday, June 15, and continuing through the 20. For application blank and full particulars, address,

F. E. Sharp, Sec'y,
Port Huron, Mich.

### MICHIGAN LICENTIATES PLEASE NOTICE.

In compliance with Section 5 of Public Act No. 183 of 1913, which is as follows:

> "Every registered dentist shall, on or before the first day of May of each year, except the one in which he is registered, pay to the secretary of the board of dental examiners, a license fee of one dollar. The year for which a fee shall be paid shall begin on October first, following the May when it becomes due, and end the succeeding September thirtieth. In case any person defaults in paying said fee, his license may be revoked by the board of dental examiners on thirty days' notice in writing from the secretary, unless within said time said fee is paid."

Notices will be mailed to all dentists registered in Michigan, to their last known address, on or before April 15, 1914. Failure to receive such notice will not be an exemption or an excuse for non-payment. In such cases all persons should notify the secretary, giving their correct address. This also applies to all those living outside the State.

Respectfully,

F. E. SHARP, Sec'y,

Port Huron, Mich.

### NATIONAL DENTAL ASSOCIATION.

The 1914 session of the National Dental Association will be held in Rochester, N. Y., July 7 to 10, 1914. The Local Committee has selected the Powers Hotel as headquarters and has made the other necessary arrangements for a large attendance.

This is the first meeting of the Association under the re-organization and the House of Delegates, the governing body, will meet at 10:30 A. M., July 8.

The officers and committees are expecting to present an exceptionally interesting programme, the details of which together with the other arrangements, will appear in the later Journals and the next number of our Official Bulletin.

HOMER C. BROWN, President,
Columbus, Ohio.
OTTO U. KING,
General Secretary,
Huntington, Ind.

---

### AMERICAN MILLER MEMORIAL.

To the Dental Profession of America.

The committee appointed by the Ohio State Dental Society at the 1909 meeting, for the purpose of raising funds for an American Memorial to the late Dr. W. D. Miller, desire to make the following report:

Funds have been received from the following states:—Alabama, $25.00; Arizona, $25.00; Arkansas, $50.00; California, $60.00; Colorado, $82.00; Connecticut, $50.00; Georgia, $60.00; Illinois, $531.00; Iowa, $200.00; Indiana, $75.00; Kansas, $134.50; Kentucky, $105.00; Maine, $25.00; Massachusetts, $100.00; Michigan, $300.00; Minnesota, $100.00; Missouri, $100.00; Montana, $15.00, Nebraska, $100.00; New Hampshire, $25.00; New Mexico, $25.00; New York, $125.00; Ohio, $1303.00; South Carolina, $25.00; North Dakota, $50.00; South Dakota, $15.00; Oklahoma, $31.00; Oregon, $50.00; Pennsylvania, $20.00; Tennessee, $50.00; West Virginia, $25.00; Washington, $50.00; Wisconsin, $25.00; Wyoming, $10.00; Texas, $50.00; Utah, $14.00;

Vermont, $20.00; Virginia, $50.00; Total, $4300.50; Interest on this fund to Dec. 1, 1913, amounts to $382.94, making a total in the hands of the treasurer, Dr. Weston A. Price, $4683.44. Florida and Mississippi have each voted $50.00 but the amounts are not in the treasurer's hands at this date.

The Memorial will consist of an 8 ft. bronze statue of Dr. Miller mounted on a 7 ft. granite pedestal and be placed in the lawn of the Public Library, the most appropriate site in the City of Columbus, the capitol of Dr. Miller's native state. Suitable tablets will be prepared and it is the desire of the committee to state on one that the monument is erected by funds from every state in the Union. If your state is not represented in the above list, we want your co-operation in placing it there.

It is hoped that sufficient funds ($5500.00) will be in the treasury that steps can be taken at once towards the construction of this memorial, that it may be finished and ready for unveiling at the 1915 meeting which will be the 50th anniversary of the Ohio State Society. The valuable co-operation of the Honorary Committees in the several states is hereby acknowledged; they have made this memorial a reality.

Other professions have done honor to their distinguished dead, let us do the same for Dr. Miller, whose life was one of unselfish devotion to the Scientific advancement of dentistry.

Respectfully,

Edward C. Mills, Chairman,
J. R. Callahan,
S. D. Ruggles,
Committee.

Columbus, Ohio, April 7, 1914.

